# The association between continuity of care and readmission to hospital in patients with severe psychosis

**DOI:** 10.1007/s00127-016-1287-3

**Published:** 2016-10-25

**Authors:** Stephen Robert Puntis, Jorun Rugkåsa, Tom Burns

**Affiliations:** 1 Department of Psychiatry, University of Oxford, Warneford Hospital, Oxford, OX3 7JX UK; 2 Health Services Research Unit, Akershus University Hospital, Lørenskog, Norway

**Keywords:** Continuity of care, Community mental health, Psychosis, Readmission

## Abstract

**Purpose:**

Providing good continuity of care to patients is considered a vital component of community mental health services, but there is limited evidence that it is associated with good outcomes. We measured service use and a multidimensional concept of continuity of care in 323 patients who were to be discharged from hospital following compulsory treatment for psychosis to investigate the association between continuity and rehospitalisation.

**Methods:**

We conducted a 36-month prospective cohort study of the patients recruited to the Oxford Community Treatment Order Trial (OCTET). We collected data from medical records on eight previously operationalized measures of continuity. We conducted regression analyses to determine the association between these measures and readmission to hospital, time to readmission, and the number of days spent in hospital.

**Results:**

Almost two thirds (*n* = 206, 63.8%) of patients were readmitted. Patients were seen frequently, with a mean of 2.9 (SD = 2.47) contacts a month throughout the follow-up. Less frequent contact was significantly associated with lower odds of rehospitalisation and fewer days in hospital. More changes in the patient’s care coordinator were associated with more time in hospital. Patients who had a higher proportion of clinical correspondence copied to them spent fewer days in hospital.

**Conclusion:**

Patients with severe and relapsing psychotic illness are seen frequently and consistently in community mental health services. Higher levels of patient contact could be a response to the severity of illness rather than a marker of quality of care. Using a simple linear interpretation of contact frequency as a measure of continuity of care in this patient group may be of limited value in modern services.

## Introduction

Continuity of care can broadly be defined as a process of delivering care to an individual patient over time which is perceived by both the patient and care providers as comprehensive, consistent, and connected. It is considered a cornerstone of modern health care provision and is included as an indicator of quality of care in national and international health policy [1, 2]. Patients and professionals endorse the importance of continuity of care, and discontinuity of care is cited as a major source of patient dissatisfaction and disengagement [3, 4]. Despite the importance placed on providing continuity, its definitions differ. There is consensus, however, that it is a multidimensional construct. The eight-dimensional definition of Freeman et al. comprising experienced, flexible, cross-boundary, information, longitudinal, relational, long-term, and contextual continuity is an influential example [5].

Evidence for associations between continuity of care and outcomes in mental health remains limited [6, 7]. We recently conducted a systematic review investigating the association between continuity of care and patient outcomes in mental health [8]. There were conflicting results for all of the most frequently examined outcomes including hospitalisation, symptom severity, social functioning, and service satisfaction. We identified methodological limitations in the studies including small sample sizes, short follow-up durations, and poorly controlled cohorts. Persisting variation in how both continuity and outcomes are measured also prevented meaningful comparisons between studies.

This study formed part of the Oxford Mental Health Coercion Research Programme and utilized the sample from the OCTET trial. This was a multi-centre randomised control trial (RCT) testing the effectiveness of community treatment under compulsion by randomising patients to either Community Treatment Order (CTO) or to voluntary treatment via short term Section 17 leave [9]. We prospectively followed the OCTET cohort for 36 months to investigate patterns of service utilization and continuity of care in this population who had been considered for a CTO because of their high likelihood of disengaging from services and risk of fragmented care. As no outcome or service differences were found between the arms in the trial, here we examine both as a single sample.

We address two research questions. First, what are the patterns of service use and continuity of care in this group of patients? Second, is there an association between continuity of care and readmission to hospital, time to readmission, or number of days in hospital? In addition, we use our results to examine continuity of care as it is currently operationalised in mental health research.

## Methods

### Sample and data collection

The 336 randomised patients in the OCTET trial were recruited from 32 mental health trusts across southern and central England. The recruitment procedure has been described in detail [9, 10]. In short, recruitment took place between 10 November 2008 and 22 February 2011 and patients were followed up for 36 months. To be eligible, the patient had to be: between the ages of 18–65 years; diagnosed with psychosis; currently detained in hospital involuntarily; considered by their clinicians as a candidate for CTO; and able to give informed consent to take part in research.

This study excluded patients who were inpatients throughout the 36 months as there would be no community service use to measure.

We used a prospective observational design and ethical approval was granted by the Staffordshire National Health Service (NHS) Research Ethics Committee (reference 08/H1204/131). Demographic data were collected via a combination of medical records and patient interview at baseline. Follow-up data were collected at 36 months by independent researchers from medical records. Details of the collection and management of the data is described elsewhere [10].

### Measures

#### Baseline measurements

Socio-demographic information and severity of psychiatric symptoms were collected at baseline. Socio-demographics included age, gender, ethnicity, country of birth, years of education, marital status, diagnosis, duration of illness, and number of past hospitalisations. Severity of symptoms was measured with the Brief Psychiatric Rating Scale (BPRS) [11].

#### Continuity of care measures

We used components from the Experiences of Continuity of Care and Health and Social Outcomes study (ECHO) [12]. ECHO operationalised the eight-dimension definition of continuity of care produced by Freeman et al. [5] and produced a 20 component, seven-factor model of continuity of care (Table 1). We utilized eight of these components in our study (Table 1). Twelve of the ECHO components were excluded for the following reasons: the Camberwell Assessment of Needs (CAN) number of met needs, CAN total level of needs met by informal carers, CAN total level of needs, and the proportion of needs met were excluded as we consider them to be outcomes of continuity of care rather than the process of continuity of care. The scale to assess the therapeutic relationship (STAR) was excluded as the therapeutic relationship is best considered a process that can lead to better continuity of care but is not a component of continuity of care (Adair, personal communication). Two components were excluded as they required patient interview (CONTINU-UM, any user-rated breaks in care). Four were excluded, after piloting the data collection process, as they could not be reliably ascertained from medical records (contacts with primary care professionals, CPA copied to GP and user, number of agencies used in previous year, attendance at day centre or hospital). Had a transition was excluded as all patients in this study by default had at least one transition as they were all discharged from hospital after being recruited.

**Table 1:** ECHO continuity of care factor structure and components

Factor	Factor name	Description	Components (*later omitted*)^a^
1	Experience and relationships	High experienced continuity, a good therapeutic relationship, a greater proportion of needs met, and not having a user-rated break in care	*CONTINU-UM* ^b^ *STAR total score—any professional* ^c^ *Proportion of needs met* *Any user-rated breaks in care*
2	Regularity	Being seen more frequently by staff from fewer different non-medical disciplines	Average gap between face-to-face contacts Gaps of 2 months or more Non-medical input spread
3	Meeting needs	High level of need, high number of met needs, and CPA copied to GP and user	*CAN total level of needs* ^d^ *CAN number of met needs* ^d^ *CPA copied to GP and user* ^e^
4	Consolidation	Having contact with fewer different agencies and not seeing primary care professionals	*Number of agencies used in previous year* *Contacts with primary care professionals*
5	Managed transitions	Having no transition, having a transition and it was documented, or having a transition that was undocumented	*Had a transition?* Documented transition
6	Care Coordination	Having a designated care coordinator, having no psychiatrist or more than two, and fewer needs met by informal carers	Designated care coordinators Designated psychiatrists *CAN total level of needs met by informal carers* ^d^
7	Supported living	Living in supported accommodation, attending day care, and having more letters copied to the user	Supported accommodation *Attendance at day centre or hospita*l Proportion of letters sent by CMHT or copied to user

^a^Items in italics were not collected in our study, for reasons given in text

^b^CONTINU-UM is the ECHO study Continuity of Care User Measure

^c^STAR is the Scale to Assess Therapeutic Relationships in Community Mental Health Care

^d^CAN is the Camberwell Assessment of Need

^e^CPA is the Care Programme Approach

The eight remaining ECHO components were measured at 36 months. These were: average gap between face-to-face contacts, gaps of two months or more, non-medical input spread (number of different professions seen), number of designated care coordinators, number of designated psychiatrists, supported living, documented transitions, and proportion of documents copied to user. These are described in full in Table 2.

**Table 2:** ECHO operationalisation components used in this study and their descriptions

Name of continuity measure	Description of continuity measure
Average gap between face-to-face contacts	*Definition* The mean number of days between each face-to-face contact for a patient and member of their community team *Rationale* The regularity with which a patient is seen contributes to the management of the patient’s symptoms and a continuing relationship between patient and community team
Gaps of two months or more	*Definition* The number of instances when 60 days or more passed between a successful face-to-face contact and the next such contact *Rationale* Discontinuities in care, such as a break in treatment, may indicate a breakdown in the relationship between patient and team
Number of professions	*Definition* The number of different non-medical professions the patient has been in contact with. Each profession was only counted once, and multiple members of the same profession could potentially see the same patient *Rationale* This component has been reconceptualised from the ECHO factor as modern mental health services are more comprehensive and designed to address many more of a patient’s needs. Greater numbers of professions seen represents more comprehensive care
Number of designated care coordinators	*Definition* The total number of care coordinators (also known as key workers) the patient was assigned to during the follow-up period *Rationale* A change in care coordinator indicates a discontinuity in the relationship between patient and community team
Number of designated psychiatrists	*Definition* The total number of consultant psychiatrists responsible for the patient’s care during the follow-up period *Rationale* A change in psychiatrist indicates a discontinuity in the relationship between patient and community team
Supported living	*Definition* Whether or not the patient was discharged to supported accommodation from their index hospitalisation *Rationale* It is an operationalisation of Freeman and colleagues’ *contextual continuity*, the social context reflected in a patient’s living situation and daily activities
Documented transitions	*Definition* Any documented referral to another agency measured as a binary yes or no variable. A documented transition is a referral which is supported by a referral letter or email with information about the patient, and recorded in patient notes. Divided into seven categories: Other mental health team; psychological services; drug and alcohol services; social care (e.g. drop in centre, day centre; support group); individualised social support and advice (e.g. money management service; individual return-to-work advisor); specialist medical (e.g. cardiology department, neurologist); and other *Rationale* Documented transitions represents Freeman and colleagues' *continuity of information*, the information transfer which follows the user
Proportion of documents copied to user	*Definition* The proportion of all letters containing information about the patient’s care sent by the CMHT which are either addressed to the patient or the patient has been copied into. Split into three categories: having 0% of letters copied to the patient; having 1–50% of letters copied to the patient; having 51–100% of all letters copied to the patient *Rationale* This component is an operationalisation of continuity of information between patient and community team

### Outcomes

We used three different measures of hospitalisation as outcome measures in this study: readmission to hospital, time to readmission, and number of days in hospital. Readmission to hospital was a binary measure (yes/no) of whether a patient was readmitted (voluntarily or involuntarily) to hospital at any point during the 36-month follow-up. Time to readmission was defined as the number of days between discharge from index admission and readmission or the end of study. Number of days in hospital was calculated as the total number of days the patient spent in hospital from readmission to the end of follow-up. Patients who were not readmitted were recorded as spending zero days in hospital.

### Analysis

We completed the statistical analysis plan before commencing data analysis. All analyses were performed using SPSS version 20. All predictor variables and outcome measures were examined using plots and graphs to check the distribution of data and identify outliers. We conducted regression analyses to investigate associations between the eight continuity measures and each of the three hospitalisation outcomes and adjusted for the baseline demographics of age, gender, ethnicity, and BPRS score. OCTET trial arm designation was not included as a covariate because there was no significant difference between the two arms in the OCTET trial, including the relative risk of admission (RR = 1.00), so including it as a covariate would not add any predictive power [13].

*Readmission to hospital* is a dichotomised outcome and logistic regression models were fitted between the predictor variables and outcome, adjusting for baseline variables. Results are reported as odds ratios (OR) with 95% confidence intervals.

*Time to readmission* is a time-to-event outcome and a proportional hazards model was fitted for each variable adjusting for baseline measures. Data were censored at the date of readmission, death, or discharge from secondary services, or end of trial, whichever occurred sooner. Results are reported as hazard ratios (HR) with 95% CIs. We conducted a sensitivity analysis using only the patients who were admitted to hospital during the trial to investigate if observed differences were due to some patients never being readmitted.

*Number of days in hospital* is a count outcome and was analysed using a negative-binomial model adjusting for each of the continuity of care measures and baseline variables. Results are reported as incidence rate ratios (IRR) with 95% CIs. A sensitivity analysis was conducted which excluded patients who were not readmitted during the study period.

#### Changes to the analysis plan

The continuity measure number of designated psychiatrists was excluded from all analyses apart from the time-sensitive analyses. During the course of the follow-up the organisation of care in England changed significantly from one where care was routinely delivered *by the same* psychiatrist when a patient was both in hospital and in the community (integrated) to psychiatrist responsibility being divided between inpatient and community care (functional). This change took place over the course of the study in all but two of the 32 recruiting mental health trusts, making the interpretation of the number of responsible psychiatrists impossible.

## Results

### Sample and baseline characteristics

The final sample included 323 of the 336 OCTET patients. Three randomised patients were excluded in the original OCTET trial as they were ineligible [9]. For this study, a further ten patients were excluded: five were inpatients throughout the 36 month follow-up period, data for four patients could not be collected (two sets of notes were destroyed in a fire at a participating Trust’s archive, the study team was denied access to one set of notes, and one set of notes was lost by an archive company), and one patient withdrew consent during the follow-up.

There were 20 deaths: 13 from natural causes, six suicides, and one accidental death from a drug-overdose. 26 patients were discharged from secondary services during the follow-up period, three of whom were discharged after moving abroad. Data for deceased or discharged patients were censored at the relevant time point.

Table 3 presents baseline socio-demographics and clinical characteristics of the sample. Mean age at baseline was 39.6 years (SD = 11.4) and 105 (33.5%) patients were female. 196 (60.7%) were White. Very few patients were married or cohabitating (*n* = 28, 8.7%), 132 (41.1%) had children. Only two patients (0.6%) were in regular employment. The majority of patients had a diagnosis of schizophrenia (*n* = 275, 85.1%). The mean number of previous hospitalisations was 6.7 (SD = 6), and time from onset of illness was a mean of 14.4 years (SD = 10.5).

**Table 3:** Baseline socio-demographic and clinical characteristics

	Total					
	Sample size	Missing data *N* (%)	*N* mean	% (SD)	Median	IQR
Age (years)	323	0	39.6	(11.4)	39	30, 47
Gender (male)	323	0	218	67.5	–	–
General education (years)	319	4 (1%)	11.9	(1.9)	11	11, 13
Ethnicity	323	0	–	–	–	–
White	–	–	196	60.7	–	–
Black	–	–	75	23.2	–	–
Asian	–	–	29	8.9	–	–
Mixed and other	–	–	23	7.1	–	–
Born in UK	322	1 (1%)	246	76.2	–	–
Married or cohabitating	321	2 (1%)	28	8.7	–	–
Children (yes)	321	2 (1%)	132	41.1	–	–
Identified carer (yes)	297	26 (8%)	111	34.4	–	–
Accommodation	309	14 (4%)	–	–	–	–
Independent	–	–	222	71.8	–	–
Supported	–	–	56	18.1	–	–
Homeless	–	–	31	10.0	–	–
Employment	322	1 (1%)	–	–	–	–
Unemployed	–	–	318	98	–	–
Voluntary/protected/sheltered	–	–	2	1	–	–
Regular employment	–	–	2	1	–	–
Duration of illness, years	313	10	14.4	(10.5)	12	6, 21
Number of past hospitalisations	302	21 (7%)	6.7	(6)	5	3, 8.3
BPRS total score	302	21 (7%)	38.7	(11.4)	36.5	32, 45
GAF total score	302	21 (7%)	38.7	(9.5)	39	30, 46.3

### Patterns of service use

#### Readmission and hospital use

As shown in Table 4, almost two thirds of patients were readmitted during the 36 months (*n* = 206, 63.8%). Patients spent a median of 107 (IQR = 31, 261) days in hospital over the three year follow-up although this was highly variable (mean = 192.4, SD = 236.6). The time to readmission was also highly variable, with a median of 539 (IQR = 192, 1057). Including only those readmitted, the median time to readmission was 249.5 days (IQR = 100.5, 489).

**Table 4:** Patient hospitalisation outcomes, patterns of care, and continuity of care at 36 month

	Sample size	*N* Mean (SD)	% Median (IQR)
Readmitted (yes)	323	206	63.8
Number of days to first readmission from index discharge	323	586.0 (413.0)	539 (192, 1057)
Of those readmitted	206	325.3 (273.9)	249.5 (100.5, 489)
Number of days in hospital from first readmission	323	140.4 (219.6)	51 (0, 174)
Of those readmitted	206	220.1 (241.1)	122 (57.8, 285.8)
Number of successful community contacts	322	105.2 (89.0)	79.5 (46.8, 130.8)
Average gap between face-to-face contacts	321	13.4 (15.4)	9.9 (5.9, 15.2)
Number of 60 day gaps without contact	322	0.9 (1.4)	
No gaps	–	181	56.2%
1 Gap	–	68	21.1%
2 Gaps	–	28	8.7%
3 or more	–	45	13.9%
Number of care coordinators (per patient)	319	2.3 (1.3)	2 (1, 3)
1	–	109	34.2%
2–3	–	157	49.2%
4 or more	–	53	16.6%
Number of consultant psychiatrists (per patient)	320	3.7 (2.8)	3 (2, 5)
1	–	74	23.2%
2–3	–	126	39.4%
4 or more	–	120	37.5%
Number of different mental health professions seen (per patient)	323	5.4 (1.7)	5 (4, 7)
Discharged from index admission to support accommodation (yes)	322	71	22%
Number of referrals to other services	312	4.3 (3.2)	4 (2, 6)
Number of documents sent to other agencies about the patient	322	18.9 (11.5)	16.0 (11, 3)
Proportion of documents copied to user	321	0.4 (0.3)	0.4 (0.2, 0.6)

#### Community service utilization and continuity of care measures

Table 4 also presents descriptive data on community service utilization during the 36-month follow-up. Patients had a median of 79.5 (IQR = 46.8, 130.8) face-to-face contacts over the 36-month follow-up. The median number of days between face-to-face contacts was 9.9 (IQR = 5.9, 15.2). 181 (56.2%) patients did not have any 60-day periods without face-to-face contact. Patients had 2.3 (SD = 1.3) different care coordinators and 3.7 (SD = 2.8) different consultant psychiatrists responsible for their care.

Patients had contact with staff from 5.4 (SD = 1.7) different types of mental health professions. Almost all patients had seen a community psychiatric nurse (*n* = 315, 97.5%) or a consultant psychiatrist (*n* = 309, 95.7%). 80.8% of patients had seen a social worker (*n* = 261), 72.8% a support worker (*n* = 235), 71.2% a staff grade psychiatrist (*n* = 230), 41.2% an occupational therapist (*n* = 133), and 22.6% a clinical psychologist (*n* = 73). 71 patients (22%) were discharged to supported accommodation directly from their index admission.

Patients had a mean of 4.3 (SD = 3.2) referrals to other services such as crisis teams, drug and alcohol services, or a mental health charity. For just under two thirds (mean = 0.6, SD = 0.4) of these referrals the referral letter was included in the patient’s records. Patients’ had a mean of 18.9 (SD = 11.5) documents sent by their community team to others involved in their care (this included letters to the patient themselves) and copies of 42% of these documents had been forwarded to the patient.

### Associations between continuity of care measures and outcomes

Table 5 presents the multivariate associations between continuity of care measures, age, gender, ethnicity, and BPRS score with readmission to hospital, time to readmission, and number of days in hospital.

**Table 5:** Multivariate associations between continuity of care variables, age, gender, ethnicity and symptom severity and readmission, time to readmission, and number of days in hospital

Measure	Readmission		Time to readmission		Number of days in hospital	
	OR (95% CI)	*p* value	HR (95% CI)	*p* value	IRR (95% CI)	*p* value
Average gap between face-to-face contacts	**0.956 (0.922–0.990)**	**0.012**	0.996 (0.989–1.003)	0.312	**0.966 (0.956–0.976)**	**0.000**
Number of 60 day gaps without contact	1.154 (0.897–1.484)	0.266	**0.597 (0.481–0.743)**	**0.000**	0.904 (0.810–1.010)	0.073
Number of different mental health professions seen	1.056 (0.776–1.436)	0.730	**0.848 (0.761–0.945)**	**0.003**	**0.861 (0.743–0.997)** ^**a**^	**0.046**
Number of care coordinators	1.154 (0.930–1.433)	0.193	**0.541 (0.435–0.673)**	**0.000**	**1.157 (1.053–1.271)**	**0.002**
Number of psychiatrists	–	–	0.923 (0.777 – 1.097)	0.363	–	–
Discharged from index admission to support accommodation (yes)	0.673 (0.355–1.273)	0.223	1.053 (0.717–1.544)	0.794	1.014 (0.743–1.385)	0.929
Any referral documented (yes)	1.631 (0.963–2.761)	0.069	1.138 (0.832–1.557)	0.418	**1.612 (1.242–2.091)** ^**a**^	**0.000**
Proportion of documents copied to user (0%)	–	–	–	–	–	–
1–50%	0.951 (0.404–2.240)	0.908	**0.272 (0.187–0.398)**	**0.000**	**0.561 (0.372–0.847)**	**0.006**
51–100%	0.794 (0.324–1.947)	0.615	**0.335 (0.223–0.503)**	**0.000**	**0.480 (0.315–0.731)**	**0.001**
Age (years)	0.984 (0.961–1.008)	0.191	0.989 (0.976–1.003)	0.126	0.992 (0.981–1.004)	0.207
Gender	0.978 (0.552–1.732)	0.939	0.873 (0.625–1.219)	0.424	1.049 (0.788–1.398)	0.741
Ethnicity (white)	–	–	–	–	–	–
Black	0.945 (0.511–1.749)	0.858	0.792 (0.555–1.131)	0.199	0.917 (0.672–1.251)	0.586
Asian	**0.283 (0.103–0.783)**	**0.015**	**0.418 (0.190–0.919)** ^**a**^	**0.030**	**0.285 (0.175–0.459)** ^**a**^	**0.000**
Other	0.989 (0.371–2.634)	0.982	1.603 (0.190–0.919)	0.111	**1.786 (1.096–2.909)** ^**a**^	**0.020**
BPRS score	1.020 (0.996–1.044)	0.107	1.012 (0.999–1.025)	0.075	**1.011 (1.000–1.023)** ^**a**^	**0.046**

Items in bold are significant at the level shown in the table

^a^Items no longer significant for sensitivity analysis on only readmitted patients

#### Readmission to hospital

The multivariate model statistic for the predictor variables and readmission to hospital was significant (*n* = 289, *x* ^2^ = 35.96, *p* = 0.001). A longer average gap between face-to-face contacts was significantly associated with lower odds of being readmitted (*p* = 0.012). Asian ethnicity was also associated with significantly reduced odds of being readmitted in comparison to White ethnicity (*p* = 0.015).

#### Time to readmission

Having more 60-day gaps between contacts was associated with longer time to readmission (*p* < 0.001), as was having seen more professions (*p* = 0.003) and having more changes in care coordinator (*p* < 0.001). Having no documents copied to the patient was associated with a shorter time to readmission in comparison to having up to half (1–50% of documents, *p* < 0.001) or more than half (51–100%, *p* < 0.001) of documents copied to the patient. Being of Asian ethnicity was also associated with a longer time to readmission in comparison to being White (*p* = 0.030).

The sensitivity analysis including only readmitted patients showed that the number of 60-day gaps, number of different professions, changes in care coordinator, and proportion of documents copied to user all remained significant. Being of Asian ethnicity was no longer associated with a longer time to readmission in comparison to being White.

#### Number of days in hospital

Having a larger average gap between face-to-face contacts (*p* < 0.001) and having contact with more professions (*p* = 0.046) were associated with fewer days in hospital. Having no documents copied to the patient was associated with more days in hospital in comparison to having up to half (*p* = 0.006) or more than half (*p* = 0.001) of documents copied to the patient. Having more changes in care coordinator (*p* = 0.002) and having any referral documented were also associated with more days in hospital. In comparison to White ethnicity, being Asian was associated with fewer days in hospital (*p* < 0.001), whilst the Other category was associated with spending more days in hospital (*p* = 0.020).

The sensitivity analysis including only patients readmitted found that the average gap between face-to-face contacts (*p* = 0.032), changes in care coordinator (*p* = 0.021), and number of documents copied to user at 1–50% (*p* = 0.014) and 51–100% (*p* = 0.002) were still significantly associated with number of days in hospital. The number of professions met, having a referral documented, BPRS, and ethnicity were no longer significantly associated with number of days in hospital.

## Discussion

We have described the patterns of service use and continuity of care in a sample of patients with severe psychosis and regular inpatient use, and tested for associations between continuity of care and hospitalisation outcomes.

### Patterns of service use

Community teams achieved a remarkable frequency of face-to-face contact with their patients, with a mean of 2.9 community contacts a month, almost one a week. Similar patterns of contact have been reported in other studies of community mental health. The UK700 trial, which compared standard care with intensive caseload management in a similar group of patients, found a mean contact frequency of 2.4 a month [14]. Studies using different methodologies (such as self-report) and shorter timescales (3–6 months) report between 2 and 4 contacts a month [15–17].

We also observed that this level of contact with patients was maintained over the 36 month follow-up. More than three-quarters of the patients (*n* = 249, 77.3%) had only one or no 60-day breaks in care (having one break could be due to a holiday or visit to family). Only 8% (*n* = 26) of patients were discharged from community services. This figure includes both disengagement and those well enough to no longer need community support. Thus, the data gathered from this study suggests that in England, patients with severe mental illness are seen regularly by their community teams and have few breaks in care. Our observed frequency and consistency of contact stand contrary to widely held views of poor follow-up in community mental health care.

There is a long-held consensus that patients are underserved or hard to engage in services. As our study indicates, patients across a wide range of health trusts are seen almost weekly as standard clinical practice.

### The association between regularity of contact and outcomes

We found that having more frequent face-to-face contact was associated with increased odds of readmission and longer hospital stays. This was in contrast to our expectations. This finding contradicts the commonly expressed understanding of more frequent and regular contact as an indicator of better continuity of care [8], one that would be assumed to result in better outcomes. However, increased contact may represent an appropriate response to the needs of the patient whose clinical condition was deteriorating prior to a relapse.

Variations in the amount of contact a patient receives may reflect the severity of a patient’s illness rather than the quality of the service. Using the frequency and consistency of contact as linear measures of continuity of care in such services may be of limited value unless measured in conjunction with the patient’s clinical condition.

Measuring changes in contact frequency in response to patient need may be more appropriate in this patient group as rates of relapse are high and symptoms fluctuate [18]. Measuring these variations may better represent the capability of services to provide continuity of care in response to relapse and recovery. This responsiveness is often referred to as *flexible continuity,* described by Freeman et al. as the ability “to be flexible and adjust to the needs of the individual over time” [5].

### The association between changes in care coordinator and outcomes

The primary source of continuity in many medical fields, such as primary care, is the relationship between doctor and patient. In community mental health, it is the care coordinator who has most frequent contact with the patient and our results confirm this.

Having fewer changes in clinician has been identified as an indicator of good continuity of care [19]. Patients may benefit from stability in their relationships with their community mental health team (CMHT) in a number of ways. Long-term patient-clinician relationships are believed to contribute to trust [20] and provide a point of stability [3]. Patients with schizophrenia who have a positive relationship with their care coordinator have also been shown to have better medication adherence than those who do not [21, 22]. They also have been found to have fewer hospitalisations, and improved symptom levels [21, 22].

We found that more frequent changes in care coordinator were associated with longer hospital stays. This is the first time this association has been demonstrated. Three previous studies did not find this association [23–25].

Patients with more changes in care coordinator also had longer than average time to readmission. However, this result may be misleading and reflect a time-dependant bias. Patients with longer time to readmission (or who were not readmitted) had more days in the community at risk of a change in care coordinator than patients who were readmitted early. This bias exists in the opposite direction for the association with number of days in hospital. Patients who spent *more* days in hospital had fewer days in the community to accrue changes in care coordinator. Due to this bias, one would expect to find fewer changes associated with more days in hospital. However, we found having more changes in care coordinator was associated with more days in hospital, which suggests a strong effect of changes in care coordinator (poor continuity) being disruptive to good community care.

### The association between copying of correspondence and outcomes

Most patients wish to be engaged with, and informed about, their treatment [26] and patients who receive information about their care report being more satisfied than those who do not [27]. We found that the practice of involving patients by sending them copies of clinical letters was associated with a reduced likelihood of early readmission and fewer hospital days. Including patients when disseminating information may improve continuity of care in two ways. First, patients may benefit directly through improving their understanding of their condition and its management. Second it may serve to foster trusting relationships.

This observed association may also have been due to other factors, such as patients’ clinical condition or accommodation status. Patients who lack insight and who are difficult to engage with may not want correspondence from the community team. Similarly, in this group of high-risk patients, accommodation arrangements can often be fluid, making written communication difficult.

### Ethnicity and hospitalisation outcomes

We observed an association between Asian ethnicity and reduced odds of readmission, longer time to readmission, and fewer days in hospital. The sensitivity analysis using only readmitted patients showed that this effect was most likely due to Asian patients being readmitted less often. Evidence on ethnicity as a predictor of hospitalisation is mixed. Older reviews find that Asian patients are more likely to be readmitted than Whites [28], whilst more recent reviews find no difference [29–32]. Our study was not designed to examine possible reasons behind this association, and therefore it is unclear why Asian participants in our study were readmitted less often.

### Measuring continuity of care

We had initially aimed to replicate the ECHO methodology in full as it is one of only two previously published multidimensional operationalisations of continuity of care in mental health research (the other being from Adair and colleagues). However, we had to exclude 12 of the 20 ECHO measures and then lost one further measure due to service changes during the study. This illustrates two key difficulties when measuring continuity of care. First, the information that services routinely record changes over time. Therefore, what may have been simple to collect in previous studies may not be possible in new studies and vice versa. Second, changes in how services are arranged may also affect the applicability of measures.

## Limitations

This study was an exploratory investigation and the findings should be interpreted with due caution. We used a fixed sample which may have been underpowered to find differences. Casual inferences cannot be made and the relationships found could be mediated by other variables not measured. We could not control for type of community team and it is possible that patients in different services may have received different levels of continuity. Whilst we collected data on a patient’s community team, patients were transferred between services often and many services went through restructuring during the study which made any meaningful analysis of these differences impossible. Finally, we recruited a group of patients who were very ill and required intensive community support, and therefore our findings may not apply to patients with less severe illness.

## Conclusion

We found that community mental health services in England maintain a high level of clinical contact with patients who are considered to have unstable psychosis and regular inpatient use. It appears community teams have developed flexible services, increasing or decreasing contact relative to illness severity. Therefore, using a simple count of intensity of contact as a linear measure of continuity of care is of limited value. The initial consensus when continuity of care was first debated in mental health was that more frequent, and more consistent, patient contact would result in better outcomes. This echoed the approach of mental health services at the time. Assuming a linear relationship between measured frequency of contact and quality of care may no longer be useful. Rather, measuring flexible continuity may give a better indication of the ability of services to provide continuity of care in this patient group.

Our study confirmed the expectation that a higher turnover of care coordinator was associated with poorer outcomes and that copying in patients to the communications about them was associated with better outcomes. These two measures are less likely to be dependent on the patients’ changing clinical condition and more likely accurate reflections of service practice and philosophy.

While continuity of care remains an important quality indicator of the process of care, this study highlights the need to create measures that are able to reflect patterns of continuity and discontinuity, rather than simply frequencies of contact.
